# Dynamic Neuromuscular Stabilization for Midlife Women with Frozen Shoulder: Clinical Effects on COP and Pain

**DOI:** 10.3390/jfmk11010045

**Published:** 2026-01-21

**Authors:** Hyeon Ji Kim, Il Bong Park, Hyun Ju Kim, Chae Kwan Lee

**Affiliations:** Department of Sports Medicine, Busan University of Foreign Studies, Busan 46234, Republic of Korea; 20255417@office.bufs.ac.kr (H.J.K.); fnjboss@bufs.ac.kr (I.B.P.); 20246204@bufs.ac.kr (H.J.K.)

**Keywords:** dynamic neuromuscular stabilization, frozen shoulder, shoulder rehabilitation, center of pressure, postural stability, midlife

## Abstract

**Objectives**: Frozen shoulder (FS) leads to pain, reduced shoulder function, and deficits in postural stability and sensorimotor control during upper-limb weight-bearing and activities of daily living tasks. This study investigated how an eight-week Dynamic Neuromuscular Stabilization (DNS) program affected Center of Pressure (COP) control and pain in midlife women with FS. **Methods**: Twenty-two midlife women with FS were randomly assigned to a DNS group (DNSG, *n* = 11) or a control group (CG, *n* = 11). The DNSG performed DNS exercises twice weekly for eight weeks, while the CG performed a dynamic stretching–based active control program. COP variables (distance, velocity, and root mean square (RMS) in the anterior–posterior (AP) and medial–lateral (ML) directions) were measured using a force platform under affected-side single-hand support with visual input and bilateral hand support with and without visual input. Pain was assessed using the Visual Analog Scale (VAS). All variables were analyzed using a two-way mixed ANOVA. **Results**: Under the affected-side single-hand support condition, a significant group × time interaction was observed for the prespecified primary outcome, ML-RMS (*p* < 0.05). Other COP variables under this condition were not significant after Holm–Bonferroni correction. Under bilateral hand-support conditions, ML-RMS remained significant after multiplicity adjustment in both visual conditions (*p* < 0.05). Pain (VAS) decreased over time in both groups, with no significant group × time interaction observed. **Conclusions**: The DNS intervention was associated with positive changes in COP-based postural control during upper-limb weight-bearing tasks in midlife women with FS. Pain decreased over time in both groups, with no significant group-by-time interaction. These findings suggest that DNS may be a potentially useful intervention for improving postural stability during upper-limb support tasks in patients with FS.

## 1. Introduction

Frozen shoulder (FS) is a disorder characterized by shoulder joint pain and limitation of range of motion [1], in which progressive restriction of movement occurs due to inflammation and fibrosis of the joint capsule [2,3]. This condition most commonly occurs after the age of 40, particularly between the ages of 50 and 60, and has been reported to show a higher prevalence in women [4]. As fibrosis and contracture of the shoulder capsule progress, pain is accompanied by limited active and passive movement of the shoulder, leading to increased stiffness [3,5]. Such painful stiffness of the shoulder causes discomfort in daily life and decreases quality of life [3,6], continuously emphasizing the need for evidence-based exercise interventions aimed at pain reduction and restoration of shoulder function and stability [7].

Particularly in midlife women, it is known that a decrease in estrogen after menopause leads to a reduction in muscle mass and deterioration in muscle fiber quality [8]. When lifestyle factors such as housework or repetitive use of the upper limbs are added, continuous mechanical loading is applied to the shoulder, exacerbating pain [9,10,11]. These mechanical factors reduce joint position sense, causing an imbalance in the normal muscle coordination that maintains joint stability [12], consequently leading to decreased postural stability of the shoulder and increased pain [13]. Therefore, midlife women can be considered a vulnerable group in which physiological and environmental factors act in combination in the development of FS.

In general, exercise interventions such as stretching are utilized in the treatment of FS [6]. These treatments are effective for pain reduction and temporary improvement of range of motion; however, reports related to the recovery of proximal–distal coordination and enhancement of neuromuscular control ability remain limited [14]. That is, a purely passive approach alone is insufficient to fully restore the intermuscular coordination and postural control functions required to maintain shoulder stability. Therefore, to fundamentally improve shoulder stability and postural control ability, an active rehabilitation approach that strengthens neuromuscular control is necessary.

Pain is an unpleasant sensory and emotional experience associated with actual or potential tissue damage, and it acts as a major cause of musculoskeletal dysfunction [15,16]. Shoulder pain is known to increase during joint movement and tends to show heightened pain sensitivity at night [17,18]. Such pain is not merely a sensory stimulus but directly affects neuromuscular control [19]. The pain in FS occurs due to inflammation and fibrosis of the joint capsule [20], and when it becomes chronic, it has been reported to cause tension in the surrounding muscles and avoidance of movement [21,22]. As a result, joint stiffness develops, and imbalances in muscle activation and abnormal movement patterns are gradually reinforced [23]. Prolonged exposure to pain decreases the accuracy of shoulder proprioception, destabilizing intermuscular coordination and postural control, which in turn may lead to weakened dynamic stability and increased postural sway, ultimately reducing the efficiency of shoulder function [13,24]. Recently, Dynamic Neuromuscular Stabilization (DNS) training has gained attention as an exercise method to restore impaired neuromuscular control [25,26].

DNS is an active rehabilitation approach that reorganizes intermuscular coordination and reactivates the movement control ability of the central nervous system through the integrated regulation of breathing, posture, and movement [27,28,29]. This neurological mechanism enhances the coordination of muscle activation between the deep stabilizing muscles and the upper-limb muscles, helping to maintain dynamic stability of the shoulder joint during upper-limb movements [28,30]. Furthermore, DNS training has been reported to be effective in improving mobility, reducing pain, and re-educating functional movement patterns in patients with joint pain [26,29]. Ultimately, by strengthening the central nervous system–based motor control ability, DNS can restore proximal–distal coordination and enable stable postural control during movement [31,32]. These characteristics complement the limitations of conventional passive therapeutic approaches and provide evidence-based intervention benefits for active neuromuscular recovery and improvement of shoulder stability.

Postural stability is considered to result from neuromuscular control and sensory integration [33,34]. Center of Pressure (COP) analysis serves as an indicator that quantitatively evaluates this postural control ability [35]. Variables such as COP velocity, distance, and root mean square (RMS) are known to reflect subtle body sway and stabilization ability [35,36]. Edouard et al. (2012) proposed a COP-based procedure in an upper-limb support position to evaluate shoulder sensorimotor control and demonstrated its feasibility and reliability in healthy adults [37]. In particular, they reported high reproducibility of COP distance and velocity variables when the lower limbs were supported up to the anterior superior iliac spine (ASIS). This suggests that these parameters may serve as useful indicators of shoulder stability and neuromuscular control. Although Edouard et al. demonstrated the validity and reliability of this measurement method in healthy adults, its applicability to pathological shoulder conditions and its utility for detecting rehabilitation-related changes remain unclear. To address this gap, the present study applied the upper-limb support–based COP measurement procedure proposed by Edouard et al. to midlife women with FS and analyzed COP changes before and after DNS intervention, thereby expanding the clinical applicability of upper-limb support–based COP assessment. This approach is meaningful in that it translates a validated COP assessment protocol from healthy adults to a pathological shoulder population and evaluates its responsiveness to a neuromuscular intervention.

In addition, previous studies have reported substantial evidence that DNS is effective in improving trunk stability [27,29]. However, no study has quantitatively verified the effects of DNS intervention on neuromuscular control and postural stability in midlife women with FS through COP analysis in a shoulder-supported posture. Therefore, the primary objective of this study was to evaluate the effects of an 8-week DNS intervention on shoulder postural stability during affected-side single-hand support, quantified by COP variables. The secondary objectives were to examine its effects on COP outcomes during bilateral hand support tasks under eyes-open and eyes-closed conditions, and on pain assessed using the Visual Analog Scale (VAS) in midlife women with FS.

## 2. Materials and Methods

This study was a randomized, parallel-group controlled trial comparing an experimental group (DNSG) and a control group (CG), with repeated measures at pre-, mid-, and post-intervention.

### 2.1. Participants

Participants were recruited from a community welfare center and Busan University of Foreign Studies located in B metropolitan city. Before enrollment, all individuals were provided with a detailed explanation of the study objectives and procedures, and written informed consent was obtained from those who agreed to participate voluntarily. The study protocol was reviewed and approved by the Institutional Review Board (IRB approval number: P01-202509-01-052), and the trial was registered in the Clinical Research Information Service (CRIS) on 20 November 2025 (registration number: KCT0011186). Although this study recruited participants from a community-based setting, all individuals included in the study had been clinically diagnosed with FS by an orthopedic or rehabilitation medicine specialist prior to participation.

The target sample size was determined using the G*Power 3.1 program (Kiel University, Kiel, Germany), with reference to a previously published randomized controlled trial that applied DNS intervention in midlife women and analyzed changes in COP as the primary outcome, and the statistical power setting used in that study was adopted. Accordingly, the sample size calculation was based on the primary COP outcome of the present study. Parameters were set at an effect size of f = 0.25, an alpha level of 0.05, and a statistical power of 0.60 [38]. The calculation indicated that at least 20 participants were required, and to account for an anticipated dropout rate of approximately 20%, 24 participants were recruited.

Group allocation was performed using a random draw method, in which participants selected numbered balls from sealed containers. According to the assigned numbers, participants were placed into either the DNSG or the dynamic stretching–based active control group. Of the 24 individuals initially assessed for eligibility, two were excluded before randomization due to health-related reasons. No participants dropped out after randomization. Therefore, all 22 randomized participants (11 per group) completed the intervention and outcome assessments and were included in the final analysis according to the intention-to-treat principle. No missing data were observed for any outcome variables; therefore, no data imputation procedures were applied. All assessments were carried out by the same trained investigator following standardized measurement protocols, and all testing conditions were maintained consistently throughout the study to minimize procedural bias. Data analysis was conducted only after the intervention and post-assessments had been completed. Due to the nature of the exercise interventions, participant blinding to group allocation was not feasible. However, hypothesis masking was applied, and participants were informed that both interventions were designed to improve shoulder function without being informed which intervention was expected to be more effective.

Inclusion Criteria

Participants were included in the study if they met all of the following criteria:

(1) Women aged between 40 and 64 years.
(2) Diagnosed with FS in the frozen stage (stage II) by an orthopedic or rehabilitation medicine specialist, with symptoms persisting for more than three months.
(3) No participation in regular structured physical activity programs, physical therapy, or sports activities involving intensive upper-limb use within the past six months.
(4) No known cardiovascular conditions or medical contraindications to moderate-intensity physical activity, based on self-reported medical history at enrollment.

Exclusion Criteria

Participants were excluded from the study if any of the following conditions applied:

(1) History of surgery involving the upper extremity or trunk within the past year.
(2) Presence of acute trauma, inflammation, or other medical conditions affecting the shoulder or upper limb.
(3) Inability to perform exercise due to severe pain or acute inflammatory symptoms.
(4) Presence of diagnosed cardiovascular, neurological, or psychiatric disorders, contraindicating moderate-intensity physical activity.
(5) Pregnancy at the time of enrollment.

**Figure 1:**
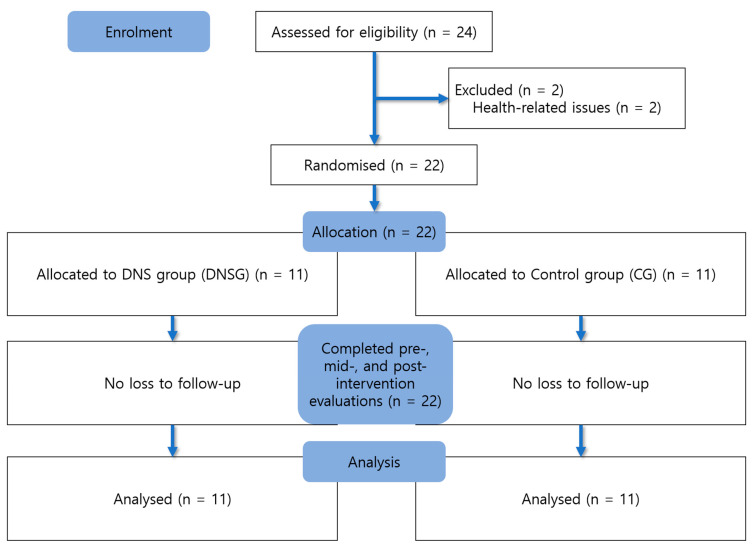
Flow diagram of participant recruitment and allocation.

In addition, to verify the baseline homogeneity between the experimental and control groups, independent *t*-tests were conducted for age, height, weight, Body Mass Index (BMI) and symptom duration. When the assumption of homogeneity of variance was violated, Welch’s *t*-test was applied. The results showed no statistically significant differences between the groups in age (t = −1.986, *p* = 0.070, df = 12.451), height (t = 2.017, *p* = 0.057, df = 20), weight (t = −0.325, *p* = 0.751, df = 11.770), BMI (t = −0.844, *p* = 0.409, df = 20), Symptom duration (t = 0.155, *p* = 0.878, df = 20). The general characteristics of the participants are summarized in Table 1, and the recruitment and group allocation procedures are illustrated in Figure 1 as a flowchart.

### 2.2. Assessment of COP and Data Acquisition

In this study, the primary and secondary outcomes were evaluated separately. The primary outcome was the COP medial–lateral (ML)-RMS measured during eyes-open affected-side single-hand support. All remaining COP variables, including other COP metrics measured under eyes-open affected-side single-hand support as well as those measured under eyes-open bilateral support and eyes-closed bilateral support conditions, and the VAS score for pain intensity were analyzed as secondary or exploratory outcomes.

In this study, the COP was analyzed to quantitatively evaluate changes in postural stability following the DNS intervention. COP measurements were obtained using a force plate (AMTI OR6, AMTI, Watertown, MA, USA) with a sampling frequency of 100 Hz. The assessment was conducted in the prone position, and to ensure consistent trunk alignment, both ASIS were positioned at the anterior edge of the treatment table. The upper limbs were extended forward to support body weight, and both palms were placed securely on the force plate.

**Figure 2:**
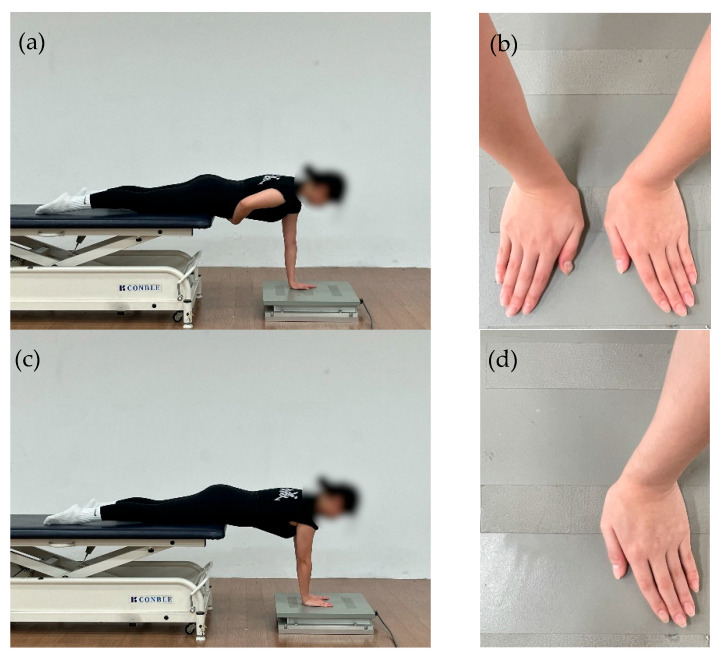
(**a**) Affected-side single-hand support position; (**b**) Hand placement on the force plate during single-hand support; (**c**) Bilateral support position; (**d**) Hand placement on the force plate during bilateral support.

The measurement procedure was adapted from the protocol of Edouard et al. [37], originally developed to assess the feasibility and reliability of shoulder sensorimotor control during upper-limb weight-bearing tasks, and was modified for clinical assessment purposes in the present study. The postural setup followed the criteria below: (1) the alignment from the shoulder joint to the wrist was adjusted to approximately 90°, (2) the wrists were maintained at approximately 15° of flexion, (3) the distance between both wrists was standardized to 4 cm, and (4) the examiner continuously monitored the participant’s posture throughout each trial to prevent compensatory trunk or scapular movements Figure 2.

The assessment was performed under three conditions: (1) eyes open with unilateral (affected-side) hand support, (2) eyes open with bilateral hand support and (3) eyes closed with bilateral hand support. The order of the measurement conditions was fixed and performed in the following sequence: eyes open with unilateral (affected-side) hand support, eyes open with bilateral hand support, and eyes closed with bilateral hand support, with all participants assessed using the same fixed order. This decision was made based on the consideration that the study population consisted of patients with FS and that visual deprivation during upper-limb weight-bearing tasks may increase postural control demands, thereby potentially affecting task stability and participant safety. Accordingly, the condition order was designed to ensure participant safety while allowing a progressive increase in task difficulty with respect to visual input and support demands in individuals with FS.

Each condition—(1) eyes open with unilateral (affected-side) hand support, (2) eyes open with bilateral hand support, and (3) eyes closed with bilateral hand support—was maintained for 30 s, with a 30 s rest period provided between conditions. One set was defined as a measurement block comprising all three conditions, and a 1 min rest period was provided between sets. Prior to the formal assessment, all participants completed a 30 s familiarization period in the same upper-limb weight-bearing posture used during testing to minimize the influence of initial learning effects. In addition, sufficient rest periods between conditions and between sets were provided to reduce the potential impact of cumulative fatigue associated with the fixed measurement order.

The raw COP signals were extracted using a force plate and smoothed using a fourth-order Butterworth low-pass filter with a cutoff frequency of 6 Hz. The displacement, velocity and RMS values were calculated for both the anterior–posterior (AP) and ML directions. COP distance in the AP and ML directions was calculated as the cumulative displacement of the COP in each respective direction over the measurement period. COP velocity in the AP and ML directions was calculated as the cumulative COP displacement in each respective direction divided by the total duration of each trial. The RMS values of COP displacement in the AP and ML directions were calculated from the force plate data and represent the variability of COP displacement around the mean COP position in each direction. Each condition—(1) eyes open with unilateral (affected-side) hand support, (2) eyes open with bilateral hand support, and (3) eyes closed with bilateral hand support—was measured five times, and the mean value of the middle three trials was used for analysis. All evaluations and data processing were performed by the same investigator to ensure measurement reliability.

### 2.3. Evaluation of Pain Intensity (VAS)

Pain intensity was evaluated using a VAS. The VAS consisted of a 10 cm horizontal line, with the left end (0 point) indicating “no pain” and the right end (10 point) indicating “extreme pain”. In this study, the VAS was used to assess current pain intensity at the time of assessment. The assessment was conducted once immediately before the COP measurement at each time point (0, 4, and 8 weeks). Participants were provided with standardized instructions and were asked to mark the point on the line that best represented the intensity of pain they were experiencing at that moment. All evaluations were performed by the same examiner under identical environmental conditions and consistent procedures to minimize external factors and ensure the reliability and objectivity of the measurements.

### 2.4. Exercise Intervention

The experimental group (EG) participated in an exercise program based on the developmental kinesiological principles of DNS. The participants performed the program twice per week for eight weeks, completing a total of 16 sessions. Each session lasted approximately 50 min and consisted of a 10 min warm-up; a 35 min main exercise phase; and a 5 min cool-down. Exercise intensity was progressively adjusted based on the number of repetitions; each movement was performed for 6 repetitions during Weeks 0–4 and 8 repetitions during Weeks 5–8. The mean adherence rate was 96%, corresponding to an average attendance of 15.5 out of 16 sessions.

Each session consisted of three phases: warm-up, main exercise, and cool-down. In the warm-up phase, participants were re-educated to form intra-abdominal pressure (IAP) and to reestablish basic trunk stabilization strategies in a supine position, based on the breathing–posture synergy. In the main exercise phase, closed-chain neuromuscular control training was performed in developmental positions, including supine, side-lying, quadruped, and modified prone positions. These exercises were designed to promote posterior–inferior stabilization of the scapula, regulate trunk stiffness, and enhance integrated strength and coordination during upper-limb support tasks [39]. The cool-down phase involved low-intensity postural readjustment and breathing exercises aimed at promoting recovery from the neuromuscular demands of the main exercise phase and stabilizing overall body tension.

**Figure 3:**
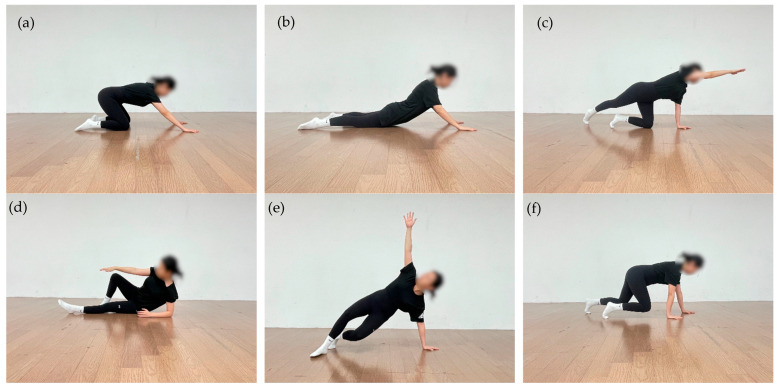
DNS exercise: (**a**) Rocking in the quadruped position; (**b**) Prone trunk extension with upper-limb support; (**c**) Contralateral hip extension in quadruped position, in which one upper limb and the contralateral lower limb are extended while trunk stability is maintained; (**d**) Oblique sitting with controlled trunk rotation; (**e**) Tall kneeling with contralateral upper-limb reach under trunk stabilization; (**f**) Quadruped with knees elevated.

The control group (CG) performed a dynamic stretching–based active control program with the same duration and frequency as the EG. Dynamic stretching is an active stretching technique that elongates the target muscle by voluntarily contracting its antagonist and repeatedly moving the associated joints [40]. This program focused on improving the mobility of the soft tissues surrounding the shoulder joint and restoring functional range of motion. Sequential stretching patterns incorporating active movements were applied to provide a control condition, ensuring general joint mobility without specific neuromuscular stabilization training. Accordingly, the CG was treated as a dynamic stretching–based active control condition primarily targeting general joint mobility. This comparison therefore contrasts DNS, which focuses on neuromuscular coordination and stabilization, with an active control intervention rather than a passive or non-treatment condition. The structure and detailed components of the training program are presented in Table 2 and Figure 3.

Outcome assessments were conducted by a single trained investigator who had received prior training in the standardized measurement protocols for all participants. The assessor was not blinded to group allocation. To minimize potential assessment bias, standardized measurement procedures were strictly applied, including identical verbal instructions, a fixed assessment order, and consistent testing environments and equipment settings across all sessions. Statistical analyses were performed after completion of all data collection according to a predefined analysis plan.

### 2.5. Statistical Analysis

All data collected in this study were analyzed using IBM SPSS Statistics for Windows, version 25.0 (IBM Corp., Armonk, NY, USA). Each variable was presented as mean ± standard deviation (SD). To examine baseline demographic characteristics between the two groups before the intervention, an independent t-test was conducted, and data normality was verified using the Shapiro–Wilk test.

A mixed-design two-way repeated-measures analysis of variance (ANOVA) was performed to evaluate the effects of the intervention. The group (experimental vs. control) was treated as the between-subject factor, whereas time (pre-, mid-, and post-intervention) was treated as the within-subject factor. When significant group × time interactions or main effects were observed, post hoc analyses with Bonferroni correction were applied to identify differences between time points.

The effect size was calculated using partial eta squared (*ηp*^2^), interpreted according to Cohen’s criteria: *ηp*^2^ = 0.01 (small), 0.06 (medium), and 0.14 (large). The level of statistical significance was set at *p* < 0.05 for all analyses.

In the present study, the pre-specified primary outcome was evaluated as the main focus of statistical interpretation. All remaining COP variables were treated as secondary or exploratory outcomes. To control for type I error inflation arising from multiple comparisons among secondary COP outcomes, these variables were considered as a single analytic family, and Holm–Bonferroni correction was applied.

## 3. Results

The analysis results of the COP variables and pain scores measured under each condition are presented in Table 3, Table 4, Table 5 and Table 6.

### 3.1. Changes in COP Under the Affected-Side Single-Hand Support Condition

The primary outcome of this study was COP ML-RMS under the eyes-open affected-side single-hand support condition. The DNSG showed a mean reduction of −0.0732 mm (95% CI −0.0888 to −0.0576), whereas the CG showed a reduction of −0.0518 mm (95% CI −0.0619 to −0.0417). The between-group difference in change (DNS − CG) was −0.0214 mm (95% CI −0.0388 to −0.0040, *p* = 0.018), favoring the DNSG.

To control type I error inflation arising from multiple comparisons among the secondary COP outcomes, Holm–Bonferroni correction was applied within the COP variable family. Although several secondary COP variables demonstrated nominally significant group × time interaction effects before adjustment, none of these variables retained statistical significance after correction. Therefore, the secondary COP findings of this study should be interpreted as exploratory rather than confirmatory.

Post hoc analyses descriptively suggested that the DNSG tended to show progressive improvements across AP- and ML-direction COP variables over time, whereas the CG generally demonstrated improvements primarily between baseline and 4 weeks, with minimal additional change thereafter. However, because these post hoc comparisons involve multiple testing and the secondary outcomes did not remain statistically significant after Holm–Bonferroni correction, these findings should be interpreted cautiously and considered exploratory.

### 3.2. Changes in COP Under the Eyes-Open Bilateral Hand Support Condition

Under the eyes-open bilateral hand support condition, three COP variables (AP-velocity, ML-velocity, and ML-RMS) showed nominally significant Time × Group interaction effects before multiplicity adjustment. To control type I error across the six COP outcomes within this condition, Holm–Bonferroni correction was applied. After adjustment, only ML-RMS retained statistical significance (adjusted *p* = 0.008), whereas AP-velocity and ML-velocity no longer met the corrected significance threshold.

### 3.3. Changes in COP Under the Eyes-Closed Bilateral Hand Support, Condition

Under the eyes-closed bilateral hand support condition, AP-velocity and ML-RMS demonstrated nominally significant Time × Group interaction effects before multiplicity adjustment, whereas AP-distance, AP-RMS, ML-distance, and ML-velocity were not significant. To control for type I error inflation across the six COP outcomes within this condition, Holm–Bonferroni correction was applied. After adjustment, only ML-RMS retained statistical significance (adjusted *p* = 0.012), whereas all other COP outcomes no longer met the corrected significance threshold.

### 3.4. Changes in Pain Indicator (VAS)

The VAS scores satisfied the assumption of sphericity and were analyzed using the original degrees of freedom.

The Time × Group interaction showed an effect size of *ηp*^2^ = 0.081, while the main effect of Time demonstrated an effect size of *ηp*^2^ = 0.756.

## 4. Discussion

This study aimed to analyze changes in the COP during upper-limb weight-bearing postures in midlife women with FS and to examine whether a DNS intervention is associated with shoulder stability and pain (VAS). The primary outcome of this study was COP ML-RMS measured during the affected-side single-hand weight-bearing posture, whereas the secondary outcomes included the remaining COP variables under the same condition, as well as COP measured during bilateral hand support under eyes-open and eyes-closed conditions and pain (VAS).

The analysis revealed a significant between-group difference in COP ML-RMS during the affected-side single-hand support posture, which was the primary outcome of this study. This finding may indicate that the DNS intervention was associated with reduced COP variability during upper-limb weight-bearing, suggesting a more consistent regulation of load control rather than generalized improvements across multiple COP metrics. Accordingly, DNS may contribute to task-specific changes in neuromuscular control during affected-side support in midlife women with FS, although broader functional or postural effects cannot be inferred from the present data.

DNS has been proposed to facilitate coordination of the scapula–upper limb–trunk in closed-chain environments and to enhance proximal stability, which may contribute to reducing fluctuations of the body’s center during load transfer. These mechanisms may be proposed as plausible explanations for the observed reduction in the primary COP variability metric (ML-RMS) following the DNS intervention. In a closed-chain condition, where the wrist and elbow are fixed, the coordination between the glenohumeral and scapulothoracic joints plays a key role in postural control [37]. DNS is interpreted to act by reorganizing joint coordination and promoting coactivation of the muscles surrounding the shoulder, which may contribute to more consistent control of the shoulder complex during weight-bearing tasks. In addition, the weight-bearing load generated during upper-limb support is transmitted through an integrated kinetic chain extending to the trunk and lower limbs, and DNS may facilitate this sequential movement, potentially allowing the load to be redistributed rather than concentrated on specific joints [41,42]. During this process, sensory feedback from each segment may support sensorimotor integration and contribute to adjustments in COP positioning in response to changes in ground reaction forces (GRF) [43,44,45].

Previous studies have reported that closed-chain exercises enhance dynamic stabilization by maintaining high joint compressive forces and joint congruency while strongly stimulating proprioceptors [46]. The upper-limb support environment provided by the DNS intervention in this study can likewise be interpreted as reinforcing such proprioceptive stimulation, thereby creating a sensory feedback–based environment in which the body may regulate load direction and balance. In this self–body-weight loading environment, muscles do not merely generate force but exhibit neuromuscular adaptations that contribute to regulating the direction of loading through the postural control system and show tendencies toward consistent regulatory patterns [47]. This process may contribute to the reduction in small-amplitude COP fluctuations, and the decreased COP observed during affected-side support after the DNS intervention can be understood not as a simple change in local muscle strength but as the result of a reorganization of the mechanical interaction among GRF, center of mass (CoM), and COP in a self–body-weight loading environment. Therefore, the reduced variability of COP following the DNS intervention may reflect changes in neuromuscular control related to load-direction regulation under weight-bearing conditions. This can be interpreted as one possible mechanism through which DNS is associated with changes in shoulder control during weight-bearing tasks in midlife women with FS.

In the secondary outcomes of this study, after Holm–Bonferroni correction, COP during both eyes-open and eyes-closed bilateral hand support demonstrated between-group differences only in ML-RMS. This finding suggests that the DNS intervention may not have induced widespread changes across all COP metrics, but rather may have been selectively reflected in indices related to mediolateral variability (ML stability). The eyes-open bilateral support task provides a wider base of support and visual feedback, making it a comparatively stable task [48]. Given this stability, DNS-related neuromuscular control changes may have been partially captured by more sensitive variability-based measures, such as ML-RMS, rather than by global velocity- or range-based COP indices. Particularly, individuals with shoulder pain tend to utilize automated compensatory strategies—such as contralateral weight shifting in stable conditions, increased visual dependence, and fixation strategies through bilateral support—which may act to reduce sway. As a result, these strategies may have limited the extent to which the effects of the DNS intervention were fully reflected in the COP outcomes. In particular, under eyes-closed bilateral hand support conditions, the absence of visual cues limits the information available for detecting postural sway, which is known to increase reliance on proprioceptive input from muscles and joints for balance control [29]. In this context, the reduction in ML-RMS observed under conditions with limited visual input may suggest that the DNS intervention was partially associated with changes in postural control mechanisms related to mediolateral variability regulation. However, as these COP changes correspond to secondary outcomes, their interpretation and clinical relevance should be approached with caution. Furthermore, additional studies with larger sample sizes and a broader range of task conditions are warranted to determine whether such changes are associated with meaningful improvements in functional performance.

The secondary outcome of pain (VAS) in this study did not show differences between groups, and both groups exhibited a decreasing trend. This indicates that changes in pain may have been influenced not by the specific characteristics of the intervention but by nonspecific factors such as the passage of time or increased general movement, which cannot be ruled out. Considering these pathological characteristics and the fact that the control intervention was an active exercise-based program, it is possible that both DNS and stretching contributed to pain reduction through shared mechanisms, including the restoration of tissue flexibility, enhanced periarticular circulation, and reduced movement avoidance. DNS may help reduce unnecessary muscle tension and compressive loading during support tasks by reorganizing the coordination of breathing, posture, and muscle activation [29,32], whereas stretching has been reported to decrease pain sensitivity by increasing joint range of motion and elongating soft tissues [49,50]. Therefore, the reduction in pain observed in this study may have been influenced not by specific effects unique to each intervention method, but rather by the shared movement stimuli and mechanical or physiological changes in the tissues provided by both interventions.

The limitations of this study are as follows:

1. The intervention period was limited to eight weeks, which may not have been sufficient to evaluate the long-term neuromuscular adaptations or sustained pain-reduction effects that may result from DNS training.
2. This study was conducted with a relatively small sample size, and the statistical power adopted in this study reflects an exploratory framework. Accordingly, the possibility of Type II error cannot be excluded, particularly for the secondary outcome variables; therefore, caution is needed when interpreting the results and generalizing the findings.
3. Pain assessment relied solely on the subjective VAS, and the evaluation of neuromuscular function was focused on COP-based analysis. The absence of objective physiological indicators such as EMG or muscle fatigue measures presents a limitation in fully elucidating the mechanisms underlying DNS.
4. The study population was restricted to midlife women, and given potential physiological differences by sex, the applicability of the findings to men may be limited.

The reduction in COP ML-RMS observed under the affected-side single-hand support condition, which was the primary outcome of this study, suggests a potential association between the DNS intervention and changes in load-related control during weight-bearing tasks. In contrast, the changes identified in the bilateral hand-support tasks, which were secondary outcomes, were limited and appeared only under specific conditions; therefore, caution is required when attempting to generalize these findings as definitive effects of DNS. In particular, the changes observed under the eyes-closed bilateral support condition may be noteworthy in that they were identified in an environment where reliance on proprioceptive input and neuromuscular regulation increases due to the absence of visual information. However, these findings should not be interpreted as direct evidence of clinical improvement or functional enhancement, and further research is required to clarify how such changes may be related to proprioceptive function and neuromuscular control. Meanwhile, the reduction in pain may be interpreted as a general physiological and mechanical effect resulting from increased movement in both the DNS and dynamic stretching–based active control group.

Future studies should analyze EMG activity of the shoulder and trunk muscles during upper-limb support tasks in conjunction with COP variables to more clearly elucidate how the DNS intervention contributes to the regulation of dynamic stability of the shoulder complex. In addition, clinical functional outcomes, including shoulder range of motion, should be incorporated to more comprehensively evaluate functional recovery following DNS intervention.

## 5. Conclusions

This study suggests that an eight-week DNS intervention may be associated with positive changes in COP-based postural control during upper-limb support postures in midlife women with FS. In particular, a significant reduction in ML-RMS was observed under the affected-side single-hand support condition, suggesting that DNS may have improved neuromuscular control among the trunk, scapula, and upper limb, thereby contributing to load transmission and postural regulation during weight-bearing tasks. Pain scores decreased over time in both groups, with no significant group-by-time interaction, suggesting a nonspecific effect of active exercise participation. Overall, these findings support the potential applicability of DNS as a useful intervention strategy for improving postural stability during upper-limb support tasks in individuals with FS, while pain reduction appeared to reflect nonspecific exercise-related effects.

## Figures and Tables

**Table 1:** Participant characteristics summary (*n* = 22).

	DNSG (Experimental, *n* = 11)	CG (Control, *n* = 11)	*p*-Value
Age (years)	56.45 ± 4.00	59.00 ± 1.41	0.070
Height (cm)	159.42 ± 4.23	156.20 ± 3.19	0.057
Weight (kg)	56.66 ± 3.87	57.99 ± 12.97	0.751
BMI (kg/m^2^)	22.34 ± 2.08	23.73 ± 5.03	0.409
Symptom duration	4.81 ± 1.25	4.72 ± 1.48	0.878
Affected shoulder, *n* (%)			
Left	4 (36.4)	5 (45.5)	
Right	7 (63.6)	6 (54.5)	

Note. Values are presented as mean ± standard deviation or number (%). DNSG, DNS group (experimental group); CG: Control group. *p*-values were calculated using independent *t*-tests or Welch’s *t*-tests, as appropriate. Categorical variables were not subjected to statistical comparison.

**Table 2:** Exercise programs for the experimental (DNSG) and control (CG) groups.

Training	DNSG (Experimental)	CG (Control)	Time/Reps
Warm-up	1. Supine diaphragmatic breathing 2. Supine cross-pattern activation	1. Standing shoulder circumduction 2. Standing arm swings in the sagittal and frontal planes 3. Standing upper thoracic extension stretch (wall-supported) 4. Side-lying shoulder internal rotation stretch 5. Standing cross-body posterior shoulder capsule stretch	10 min /8 reps
Exercise	1. Side-lying rolling 2. Rocking in the quadruped position 3. Prone trunk extension with upper-limb support 4. Contralateral hip extension in quadruped position 5. Oblique sitting with controlled trunk rotation 6. Tall kneeling with contralateral upper-limb reach under trunk stabilization 7. Quadruped Locomotion Pattern 8. Transition from Quadruped to High Side Support 9. Quadruped with knees elevated 10. Alternating upper-limb unloading in quadruped position with knees elevated	1. Standing shoulder external & internal rotation 2. Standing Scapular retraction exercise 3. Standing dynamic shoulder movement (“Y” and “T” patterns) 4. Shoulder protraction exercise in the quadruped position 5. Standing diagonal shoulder movement pattern 6. Standing Doorway pectoral stretch 7. Active shoulder flexion stretching in the supine position 8. Side-lying cross-body shoulder stretch	35 min /6 reps −8 reps
Cool-down	1. Supine resting position for deep trunk stabilization 2. Prone resting position with diaphragmatic breathing control	1. Side-lying repeated upper thoracic stretch 2. Standing Arm and shoulder swing	5 min /8 reps

Note. DNSG, DNS group (experimental group); CG: Control group.

**Table 3:** Changes in COP parameters under eyes-open affected-side single-hand support condition.

Variables	Assessment	DNSG	CG	Group × Time			Time			Post Hoc DNSG	Post Hoc CG
				*p*	F (df)	*ηp* ^2^	*p*	F (df)	*ηp* ^2^		
AP- Distance (mm)	0 Weeks	86.62 ± 14.08	86.84 ± 10.16	0.048	3.82 (1.402, 28.040)	0.160	<0.001	79.61 (1.402, 28.040)	0.799	0–4 w: 0.013	0–4 w: <0.001
	4 Weeks	66.59 ± 11.36	61.39 ± 5.45							4–8 w: <0.001	4–8 w: 0.555
	8 Weeks	49.44 ± 8.96	58.75 ± 7.79							0–8 w: <0.001	0–8 w: <0.001
AP- velocity (mm/s)	0 Weeks	2.88 ± 0.46	2.83 ± 0.39	0.041	4.04 (1.429, 28.576)	0.168	<0.001	67.103 (1.429, 28.576)	0.770	0–4 w: 0.013	0–4 w: 0.001
	4 Weeks	2.21 ± 0.37	1.96 ± 0.37							4–8 w: 0.001	4–8 w: 1.000
	8 Weeks	1.71 ± 0.28	1.97 ± 0.32							0–8 w: <0.001	0–8 w: 0.001
AP-RMS (mm)	0 Weeks	0.21 ± 0.02	0.21 ± 0.05	0.233	1.53 (1.495, 29.903)	0.071	<0.001	57.495 (1.495, 29.903)	0.742	N/A	N/A
	4 Weeks	0.15 ± 0.01	0.14 ± 0.02							N/A	N/A
	8 Weeks	0.12 ± 0.01	0.14 ± 0.04							N/A	N/A
ML- Distance (mm)	0 Weeks	87.24 ± 13.57	84.75 ± 20.41	0.238	1.49 (2, 40)	0.069	<0.001	44.004 (2, 40)	0.688	N/A	N/A
	4 Weeks	69.55 ± 10.65	58.96 ± 11.58							N/A	N/A
	8 Weeks	57.81 ± 13.82	57.59 ± 12.38							N/A	N/A
ML- velocity (mm/s)	0 Weeks	2.90 ± 0.45	2.82 ± 0.31	0.030	3.83 (2, 40)	0.161	<0.001	48.05 (2, 40)	0.706	0–4 w: 0.016	0–4 w: <0.001
	4 Weeks	2.31 ± 0.35	2.02 ± 0.33							4–8 w: 0.020	4–8 w: 0.947
	8 Weeks	1.91 ± 0.30	2.12 ± 0.15							0–8 w: <0.001	0–8 w: <0.001
ML-RMS (mm)	0 Weeks	0.20 ± 0.03	0.20 ± 0.02	0.009	5.25 (2, 40)	0.208	<0.001	104.894 (2, 40)	0.840	0–4 w: <0.001	0–4 w: <0.001
	4 Weeks	0.17 ± 0.03	0.16 ± 0.01							4–8 w: 0.002	4–8 w: 0.081
	8 Weeks	0.13 ± 0.02	0.15 ± 0.02							0–8 w: <0.001	0–8 w: <0.001

Note: Data are presented as mean ± standard deviation. DNSG, DNS group (experimental group); CG: Control group. *p*-values are based on mixed-design two-way ANOVA. F = F statistic derived from the mixed-design two-way ANOVA. *ηp*^2^ = Partial eta squared (effect size): small = 0.01, medium = 0.06, large = 0.14. Post hoc comparisons were adjusted using the Bonferroni correction. AP = anterior–posterior; ML = medial–lateral. N/A = post hoc analysis not applicable (Group × Time interaction not significant).

**Table 4:** Changes in COP parameters under eyes-open bilateral support condition.

Variables	Assessment	DNSG	CG	Group × Time			Time			Post Hoc DNSG	Post Hoc CG
				*p*	F (df)	*ηp* ^2^	*p*	F (df)	*ηp* ^2^		
AP- Distance (mm)	0 Weeks	58.32 ± 9.14	57.87 ± 13.51	0.730	0.32 (2, 40)	0.016	<0.001	44.03 (2, 40)	0.688	N/A	N/A
	4 Weeks	49.65 ± 8.24	46.13 ± 11.86							N/A	N/A
	8 Weeks	40.35 ± 7.04	38.95 ± 5.88							N/A	N/A
AP- velocity (mm/s)	0 Weeks	1.94 ± 0.30	1.89 ± 0.52	0.029	4.66 (1.377, 27.539)	0.189	<0.001	30.12 (1.377, 27.539)	0.601	0–4 w: 0.010	0–4 w: 0.001
	4 Weeks	1.58 ± 0.21	1.55 ± 0.36							4–8 w: <0.001	4–8 w: 0.942
	8 Weeks	1.23 ± 0.18	1.56 ± 0.26							0–8 w: <0.001	0–8 w: 0.001
AP-RMS (mm)	0 Weeks	0.17 ± 0.03	0.20 ± 0.04	0.590	0.54 (2, 40)	0.026	<0.001	45.94 (2, 40)	0.697	N/A	N/A
	4 Weeks	0.12 ± 0.02	0.14 ± 0.04							N/A	N/A
	8 Weeks	0.09 ± 0.01	0.13 ± 0.02							N/A	N/A
ML- Distance (mm)	0 Weeks	65.19 ± 13.62	67.20 ± 11.37	0.754	0.20 (1.471, 29.412)	0.010	<0.001	71.68 (1.471, 29.412)	0.782	N/A	N/A
	4 Weeks	45.25 ± 6.79	45.31 ± 8.75							N/A	N/A
	8 Weeks	37.86 ± 4.30	40.83 ± 3.60							N/A	N/A
ML- velocity (mm/s)	0 Weeks	2.17 ± 0.45	2.22 ± 0.74	0.557	0.59 (2, 40)	0.029	<0.001	51.15 (2, 40)	0.719	N/A	N/A
	4 Weeks	1.50 ± 0.22	1.35 ± 0.34							N/A	N/A
	8 Weeks	1.33 ± 0.22	1.23 ± 0.24							N/A	N/A
ML-RMS (mm)	0 Weeks	0.18 ± 0.03	0.17 ± 0.03	<0.001	9.69 (2, 40)	0.326	<0.001	55.94 (2, 40)	0.737	0–4 w: 0.127	0–4 w: <0.001
	4 Weeks	0.15 ± 0.04	0.12 ± 0.02							4–8 w: 0.001	4–8 w: 1.000
	8 Weeks	0.09 ± 0.00	0.12 ± 0.02							0–8 w: <0.001	0–8 w: <0.001

Note: Data are presented as mean ± standard deviation. DNSG, DNS group (experimental group); CG: Control group. *p*-values are based on mixed-design two-way ANOVA. F = F statistic derived from the mixed-design two-way ANOVA. *ηp*^2^ = Partial eta squared (effect size): small = 0.01, medium = 0.06, large = 0.14. Post hoc comparisons were adjusted using the Bonferroni correction. AP = anterior–posterior; ML = medial–lateral. N/A = post hoc analysis not applicable (Group × Time interaction not significant).

**Table 5:** Changes in COP parameters under eyes-closed bilateral support condition.

Variables	Assessment	DNSG	CG	Group × Time			Time			Post Hoc DNSG	Post Hoc CG
				*p*	F (df)	*ηp* ^2^	*p*	F (df)	*ηp* ^2^		
AP- Distance (mm)	0 Weeks	59.25 ± 9.00	57.10 ± 6.05	0.562	0.58 (2, 40)	0.028	<0.001	34.97 (2, 40)	0.636	N/A	N/A
	4 Weeks	50.88 ± 8.38	46.25 ± 8.41							N/A	N/A
	8 Weeks	42.04 ± 6.98	41.64 ± 6.44							N/A	N/A
AP- velocity (mm/s)	0 Weeks	1.97 ± 0.30	1.96 ± 0.30	0.040	3.97 (2, 40)	0.166	<0.001	55.90 (2, 40)	0.736	0–4 w: 0.024	0–4 w: 0.001
	4 Weeks	1.62 ± 0.31	1.54 ± 0.36							4–8 w: 0.002	4–8 w: 1.000
	8 Weeks	1.33 ± 0.21	1.53 ± 0.26							0–8 w: <0.001	0–8 w: <0.001
AP-RMS (mm)	0 Weeks	0.15 ± 0.02	0.18 ± 0.03	0.382	0.99 (2, 40)	0.047	<0.001	44.06 (2, 40)	0.688	N/A	N/A
	4 Weeks	0.12 ± 0.01	0.14 ± 0.01							N/A	N/A
	8 Weeks	0.10 ± 0.02	0.12 ± 0.01							N/A	N/A
ML- Distance (mm)	0 Weeks	65.97 ± 9.61	67.70 ± 8.67	0.877	0.13 (2, 40)	0.007	<0.001	99.34 (2, 40)	0.832	N/A	N/A
	4 Weeks	45.27 ± 6.48	45.07 ± 9.68							N/A	N/A
	8 Weeks	39.53 ± 6.35	40.94 ± 8.07							N/A	N/A
ML- velocity (mm/s)	0 Weeks	2.25 ± 0.20	2.24 ± 0.51	0.045	3.94 (1.378, 27.564)	0.164	<0.001	96.67 (1.378, 27.564)	0.829	0–4 w: <0.001	0–4 w: 0.001
	4 Weeks	1.51 ± 0.20	1.53 ± 0.41							4–8 w: <0.001	4–8 w: 1.000
	8 Weeks	1.12 ± 0.15	1.55 ± 0.34							0–8 w: <0.001	0–8 w: 0.001
ML-RMS (mm)	0 Weeks	0.16 ± 0.02	0.15 ± 0.02	0.002	7.51 (2, 40)	0.273	<0.001	28.06 (2, 40)	0.584	0–4 w: 0.001	0–4 w: 0.317
	4 Weeks	0.13 ± 0.03	0.13 ± 0.03							4–8 w: 0.001	4–8 w: 1.000
	8 Weeks	0.09 ± 0.01	0.13 ± 0.04							0–8 w: <0.001	0–8 w: 0.214

Note: Data are presented as mean ± standard deviation. DNSG, DNS group (experimental group); CG: Control group. *p*-values are based on mixed-design two-way ANOVA. F = F statistic derived from the mixed-design two-way ANOVA. *ηp*^2^ = Partial eta squared (effect size): small = 0.01, medium = 0.06, large = 0.14. Post hoc comparisons were adjusted using the Bonferroni correction. AP = anterior–posterior; ML = medial–lateral. N/A = post hoc analysis not applicable (Group × Time interaction not significant).

**Table 6:** Changes in VAS over time in the affected side.

Assessment	DNSG	CG	Group × Time			Time		
			*p*	F (df)	*ηp* ^2^	*p*	F (df)	*ηp* ^2^
0 Weeks	6.27 ± 1.42	5.36 ± 0.80	0.186	1.756 (2, 40)	0.081	<0.001	62.091 (2, 40)	0.756
4 Weeks	3.63 ± 1.62	3.72 ± 1.00						
8 Weeks	1.90 ± 1.37	2.18 ± 1.60						

Note: Data are presented as mean ± standard deviation. DNSG, DNS group (experimental group); CG: Control group. *p*-values are based on mixed-design two-way ANOVA. F = F statistic derived from the mixed-design two-way ANOVA. *ηp*^2^: Partial eta squared (small = 0.01, medium = 0.06, large = 0.14).

## Data Availability

The data used in this study are available upon reasonable request and will be deposited in a public repository upon publication.
